# Epidemiological investigation of goose circovirus based on a newly developed indirect ELISA method

**DOI:** 10.3389/fvets.2025.1521705

**Published:** 2025-02-13

**Authors:** Jialong Chen, Wenchang Xue, Zhanxin Yao, Chao Wang, Wanjun Zhu, He Wang, Jipei Zhang, Yi Tang, Rongchang Liu, Jidang Chen

**Affiliations:** ^1^School of Animal Science and Technology, Foshan University, Foshan, China; ^2^WANMUZHOU Biotechnology Limited Company, Foshan, China; ^3^Shenzhen Kingkey Smart Agriculture Times Co., Ltd., Shenzhen, China; ^4^College of Animal Science and Veterinary Medicine, Shandong Agricultural University, Taian, China; ^5^Institute of Animal Husbandry and Veterinary, Fujian Academy of Agricultural Sciences, Fuzhou, China; ^6^Agricultural Teaching and Research Bases, Foshan University, Foshan, China

**Keywords:** goose circovirus, capsid protein, serological detection methods, prokaryotic expression, indirect ELISA

## Abstract

Goose circovirus (GoCV) is a recently identified pathogen in geese that is known to cause slow growth, feather disorder syndrome, and immunosuppression. Infection with GoCV may increase the risk of coinfections with multiple pathogens, leading to significant economic losses in the goose industry. However, due to a lack of serological detection methods, analysis of viral nucleic acids has been widely used in GoCV epidemiological surveys, which has limited accurate monitoring of the prevalence of GoCV. In this study, we developed and optimized an indirect ELISA method based on the prokaryotic-expressed recombinant GoCV capsid protein (△Cap-iELISA). The △Cap-iELISA was then used to test 349 goose serum samples collected from Guangdong, Shandong, and Fujian provinces during 2023 and 2024. The results showed that the positive rate of GoCV antibodies in the sampled geese was 71.06%. Further analysis indicated that the positive rate of GoCV antibodies increased with the age of the geese. In conclusion, we have developed a novel iELISA method that is well-suited for large-scale clinical detection and early diagnosis of GoCV infection. Notably, a significant correlation between age and the positive rate of GoCV antibodies among geese was observed based on this newly established method.

## Introduction

Goose circovirus (GoCV) is a non-enveloped virus with a circular single-stranded DNA genome 1.8 kb in size (1), making it the smallest known animal virus. GoCV belongs to the Circovirus the genus Circovirus with in the family Circoviridae (2). Porcine circovirus (PCV), duck circovirus (DuCV), and GoCV share a similar genome structure that features three open reading frames (*ORFs*), *ORFV1*, *ORFC1*, and *ORFC2*, on both complementary DNA strands. The transcription of circoviruses is bidirectional. The sense strand encodes the replicase protein (Rep/Rep0) and the virus Capsid by *ORFV1* and *ORFC1*, respectively. In addition, the apoptosis-related protein is encoded by *ORFC2* located on the complementary strand (3, 4).

Cap protein is the main structural protein of GoCV (5). There are five major B-cell antigenic epitopes on Cap protein, which are located at 28aa–47aa, 129aa–148aa, 105aa–124aa, 156aa–175aa, and 231aa–250aa; followed by two glycosylation sites at 49aa and 141aa. proteins play an essential role in neutralizing antibodies against GoCV. In our previous sequence analysis we found that the nucleotide similarity of GoCV Cap proteins ranged from 86.3 to 98.5%, and the amino acid sequence similarity ranged from 92.4 to 100%, with the highest upper limits of amino acid sequence similarity between PCV2 Cap, BFDV Cap, and GoCV Cap being 37.09 and 31.13%, respectively (6). In addition we found that the Cap protein contains 17 arginine residues at the N-terminus, which correlates with the nuclear localization of the protein. It has been shown that a high percentage of arginine residues can greatly reduce the expression level of recombinant proteins in *E. coli* (7). This may be an important reason for the non-expression or low expression of the Cap protein of circovirus in *E. coli* (8, 9).

GoCV was firstly discovered in a flock of geese in Germany in 1999 by Soike et al. (10). Subsequently, it was reported in Hungary (11), the United Kingdom (12), Poland (13) and other countries. In China, GoCV was first detected in a flock of geese in Taiwan Province in 2003 (14). Subsequently, infected geese were also found in Zhejiang (15), Fujian, Guangxi and Guangdong (16). Based on early observations of GoCV infection and associated symptoms, the main clinical signs in geese include growth retardation, feather stunting, shedding, and feather follicular necrosis (17). These symptoms can severely affect the appearance of a goose’s carcass, which can have a significant impact on yield. However, GoCV has a low direct lethality in geese and clinical signs and lesions are not readily detectable (18). In addition, current nucleic acid-based GoCV detection methods require more time, more procedural steps, and are costly, leading to limitations in monitoring and preventing GoCV (19). Therefore, there is an urgent need for sensitive and efficient serologic methods to detect GoCV to diagnose the disease.

In this study, we report the establishment and optimization of an indirect ELISA (△Cap-iELISA) detection method that is based on coating a truncated Capsid protein (△Cap) to evaluate the seroprevalence of GoCV. A total of 349 serum samples from geese were tested in the three major goose breeding areas of Guangdong, Shandong, and Fujian provinces. The ELISA developed in this study, is suitable for large-scale clinical detection and thus provides a new method for the early diagnosis of GoCV infection. Furthermore, a notable correlation was observed between age and the positive rate of GoCV antibodies among geese, indicating an escalating trend with advancing age. The utilization of △Cap-iELISA has enriched the seroepidemiological survey data of GoCV in goose flocks, thus holding significant value for determining the current status of the GoCV epidemic and devising targeted prevention and control strategies.

## Materials and methods

### Plasmids, serum samples

BL21 (DE3) competent cells (TaKaRa, China) and the expression vector pET-30a(+) (Unibio, China) were stored at −20°C in the laboratory. The reference positive and negative sera for GoCV, validated by Western blots of the GoCV △Cap protein, were previously stored in the lab. Additionally, reference goose serum samples for specificity analysis against H5 and H7 subtypes of Avian influenza virus (AIV), Newcastle disease virus (NDV), Goose paramyxovirus (GPMV), Avian reovirus (ARV), Tembusu virus (TMUV) Goose parvovirus (GPV). Sera samples for filed study, were randomly collected from geese breeding-intensive areas in Guangdong, Fujian, and Shandong Provinces between 2023 and 2024. A total of 349 samples were obtained from goslings, meat geese, and breeding geese. There were 239 serum samples from Jiangmen, Zhaoqing, Enping, and Yangjiang in Guangdong, 60 serum samples from Hua’an City in Fujian, and 50 serum samples from Wulong City in Shandong. Serum samples mentioned above were stored at −80°C.

### Codon optimization of cap and recombinant plasmid construction

The △Cap gene (37aa-250aa) of strain GoCV/369/GD/2020 (GenBank Accession No. MT831925, referred to as GoCV/369) with the nuclear localization sequence (NLS) removed was synthesized by Genscript (Nanjing, China) using codon optimization software (GenSmart^™^ Codon Optimization) (the modified sequence is included in the Supplementary material). Two primers with restriction enzyme cutting sites were designed to amplify the synthetic *△Cap* gene: *Cap-NdeI-F* (GCTACATATGCACCACCACCACCACCACTCAAAGTACACT-3′) and *Cap-HindIII-R* (5′-AGCTAAGCTTTCATTACGGCGCCAGGCCGGT-3′). The amplified gene was subsequently cloned into the plasmid pET-30a (+) with a His tag for further expression. The resulting plasmid, termed pET30a-△Cap, underwent verification through PCR, double enzyme digestion using *NdeI* and *HindIII* enzymes from New England Biolabs (United States), and sequencing conducted by Genewiz (China).

### Expression of the recombinant cap protein in BL21 (DE3)

For protein expression, the pET30a-△Cap plasmid was initially transformed into *E. coli* BL21 (DE3) competent cells and then cultured on LB solid medium plates with 50 μg/mL of Kanamycin sulfate at 37°C. Five colonies were selected and cultured in LB medium with 50 μg/mL of Kanamycin sulfate and incubated at 37°C until the optical density at 600 nm (OD600 nm) reached 0.6 to 0.7. Following this, 0.5 mM IPTG (isopropyl-β-D-thiogalactopyranoside, Beyotime Biotechnology) was added to induce protein expression. The ultrasonic-disrupted bacterial lysates were then analyzed by 12% SDS-PAGE (sodium dodecyl sulfate-polyacrylamide gel electrophoresis). The optimal concentration of IPTG (0.25, 0.50, 0.75, or 1.00 mM) and induction time (1, 2, 3, 4, 5, 6, 7, and 8 h) were determined through a gradient test based on equal loading quantities of total protein in each group.

### Protein purification and Western blot assays

The △Cap protein was expressed and identified by Western blotting followed purification using a Ni+-agarose His-tagged protein purification kit (Beyotime, China). The concentration of the purified protein was determined using the BCA method and analyzed by SDS-PAGE. To quantify the expression level of the △Cap protein, Western blotting was performed using a mouse anti-His tag monoclonal antibody (Beyotime, China) as the primary antibody (dilution 1:1000), sheep anti-mouse IgG labeled with HRP (Proteintech, United States) as the secondary antibody (dilution = 1:10000), and BeyoECL Plus (Beyotime, China) as the chromogenic agent.

### Preparation and identification of rabbit anti-△cap polyclonal antibodies

Two 10-week-old female New Zealand rabbits were selected for the study and divided into an immunization group and a blank control group, and negative serum samples were collected using marginal ear vein blood collection prior to immunization. The experimental rabbits were immunized with purified recombinant protein via subcutaneous multipoint injection at a dose of 1 mg per rabbit. Freund’s complete adjuvant was used for the first immunization, followed by emulsification with Freund’s incomplete adjuvant for the second and third immunizations. Serum samples were collected on the seventh day after each immunization, with subsequent booster shots administered every 10 days. After three rounds of immunization, blood was collected from the heart to obtain a large amount of serum, which was then frozen at −80°C. The prepared rabbit polyclonal antibody served as the primary antibody, while sheep anti-rabbit IgG was used as the secondary antibody for Western blot identification.

### Development and optimization of the △cap-iELISA

A checkerboard titration method was utilized to determine the optimal concentration of the coated antigen and the proper serum dilution (20), Both antigen and serum were added to 96-well plates with 100 μL/well, respectively. Briefly, the purified △Cap protein was diluted with a coating solution to 0.5, 1, 2, 3, and 4 μg /mL and then coated onto a 96-well plate. GoCV-positive and negative serum samples were employed to establish the optimal working conditions at dilutions of 1:50, 1:100, 1:200, and 1:400. The HRP labeled goat anti-Duck IgG (H + L) (KPL, USA) antibody was used at different dilutions (1:1000, 1:2000, 1:5000, and 1:8000). Following color development with TMB (Solarbio, China), the reaction was stopped with ELISA stop solution (Solarbio, China). The absorbance of each well at OD450 nm was measured using a microplate reader. The dilution that exhibited the greatest difference in absorbance of P/N (i.e., positive and negative sera) at OD450 nm was determined as the optimal working dilution for testing experimental serum samples. Thirteen GoCV-negative serum samples that had been prevalidated by Western blotting and PCR were tested using the optimized iELISA reaction conditions to determine the cut-off value. The cut-off value was calculated based on the following formula: cut-off value = mean OD450 nm value +3 standard deviations (21).

### Detection of specificity and repeatability

Various anti-sera, including GoCV, AIV, NDV, GMPV, ARV, GPV, and TMUV, were employed to evaluate the antigenic cross-reactivity of the △Cap-iELISA. Six serum samples were randomly selected for intra-batch and between-batch replicates, and the mean, standard deviation, and coefficient of variation of each sample were calculated.

### Comparison of the △cap-iELISA with real-time quantitative PCR

A total of 118 serum samples were collected from geese that were suspected of being infected with GoCV (i.e., exhibiting feather growth disorder and dysplasia). The serum samples underwent △Cap-ELISA and Real-time Quantitative PCR assays simultaneously for comparative evaluation of the △Cap-ELISA test. Each sample was tested in triplicate for technical accuracy. The primers used for the Real-time Quantitative PCR detection were qGoCV-F: 5′-GGTCTGCCGATAACTGA-3′ and qGoCV-R: 5′-GGCCGACCAATCAGAACGA-3′.

### Application of iELISA for epidemiological surveys of GoCV infection

The information of sera samples for field study was mentioned previously at the begining of this section. The △Cap-iELISA method established in this study was utilized to conduct serological surveys of goose circovirus capsid protein antibodies in the aforementioned regions.

## Results

### Preparation of the △cap protein

**Figure 1:**
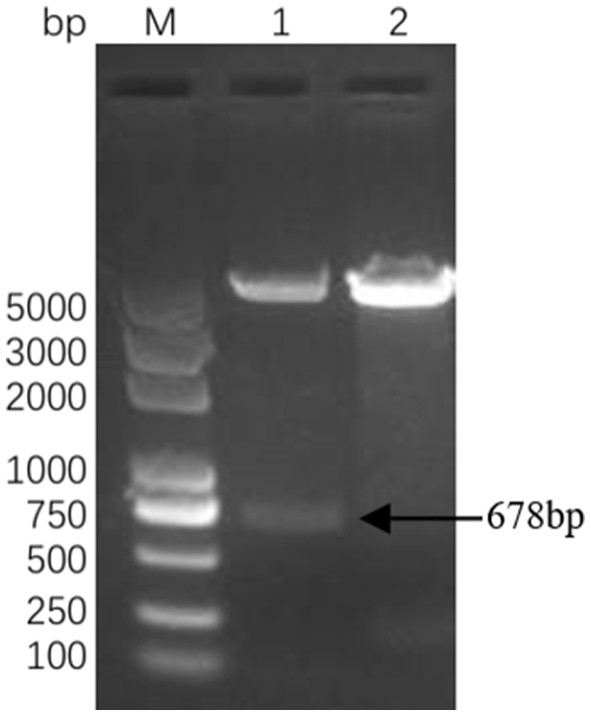
Identification of the recombinant plasmid pET-30a-△Cap. Lane M DL2000 Puls DNA marker; Lane 1 pET-30a-△Cap enzyme double digestion, Lane 2 pET-30a-(+) (*NdeI* + *HindIII*).

**Figure 2:**
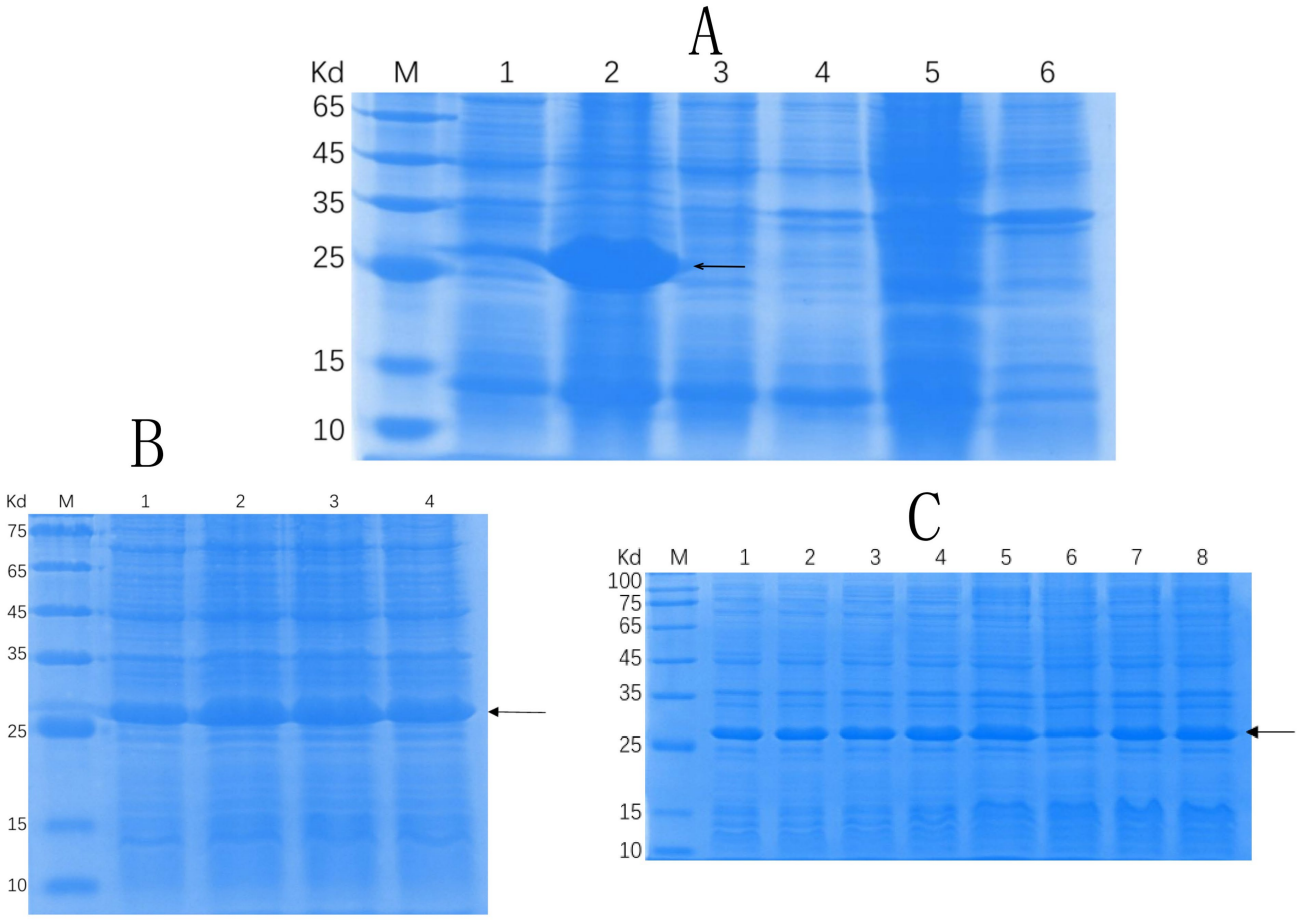
SDS-PAGE analysis of solubility and the optimization of expression conditions of the recombinant protein Cap. **(A)** SDS-PAGE analysis of △Cap protein solubility. Lane M, protein molecular quality standard. Lanes 1–2, the supernatant and precipitate after crushing and centrifugation of pET30a-△Cap-BL21 (DE3), respectively. Lanes 3–4, the supernatant and precipitate after crushing and centrifugation of pET30a--BL21 (DE3), respectively; Lanes 5–6: the supernatant and precipitate after crushing and centrifugation of BL21 (DE3), respectively. **(B)** SDS-PAGE analysis of the △Cap protein at different IPTG-induced concentrations. Lane M, protein molecular quality standard. Lanes 1–4, the samples with IPTG concentrations of 0.25, 0.5, 0.75, and 1 mM. **(C)** SDS-PAGE analysis of the △Cap protein at different induction times. Lane M, protein molecular quality standard. Lanes 1–8: Samples induced for IPTG for 1, 2, 3, 4, 5, 6, 7, and 8 h. The arrows pointed band are the expected protein.

**Figure 3:**
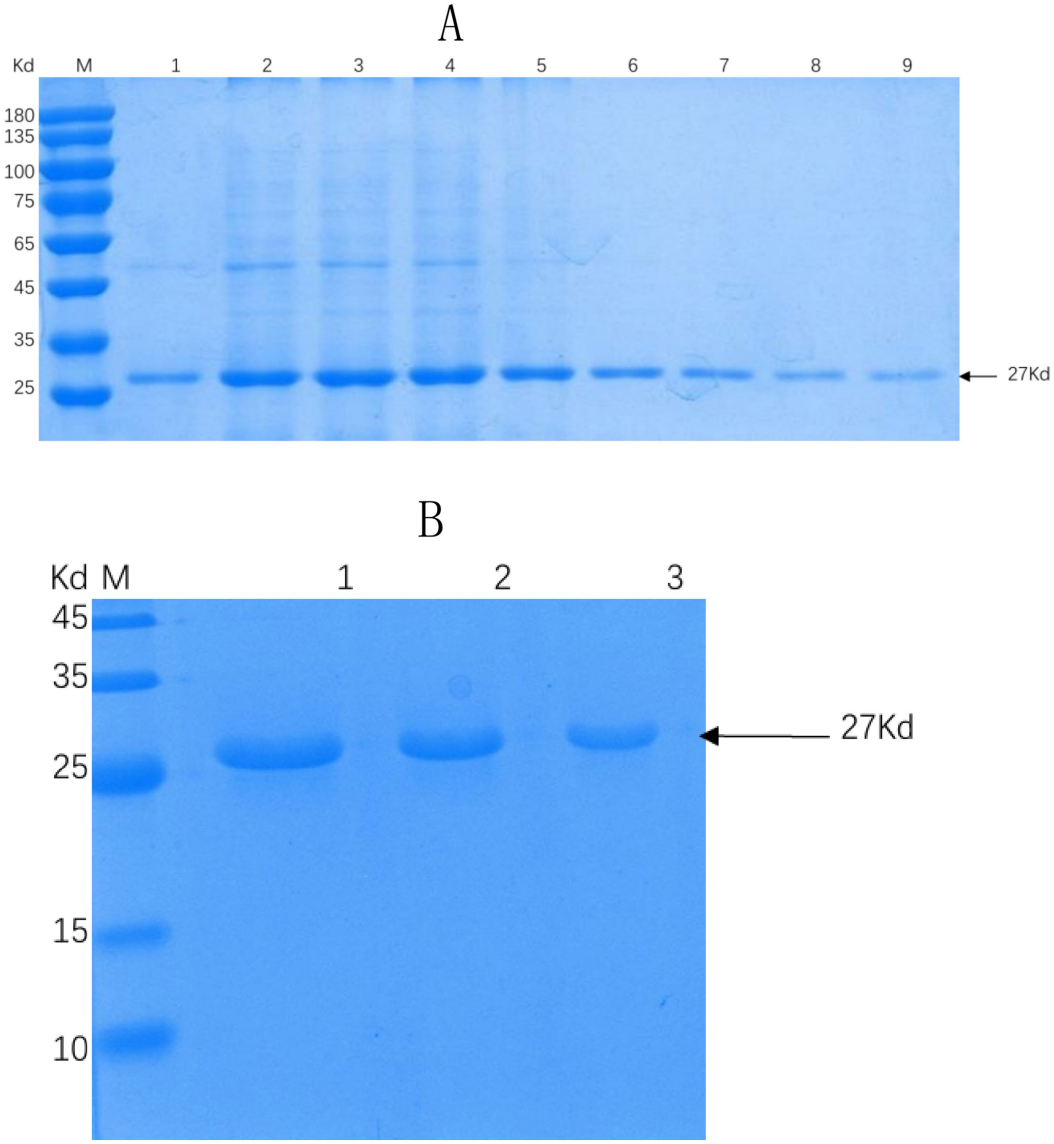
SDS-PAGE analysis of recombinant proteins under different purification conditions. **(A)** SDS-PAGE analysis of △Cap protein purified by nickel column affinity chromatography. Lane M, protein molecular quality standard. Lanes 1–9, the collected △Cap proteins at different elution concentrations. **(B)** SDS-PAGE analysis of the △Cap protein purified by gel purification protein. Lane M, protein molecular quality standard. Lanes 1–3, △Cap protein samples of different concentrations were purified by cutting rubber. The arrows pointed band are the expected protein.

**Figure 4:**
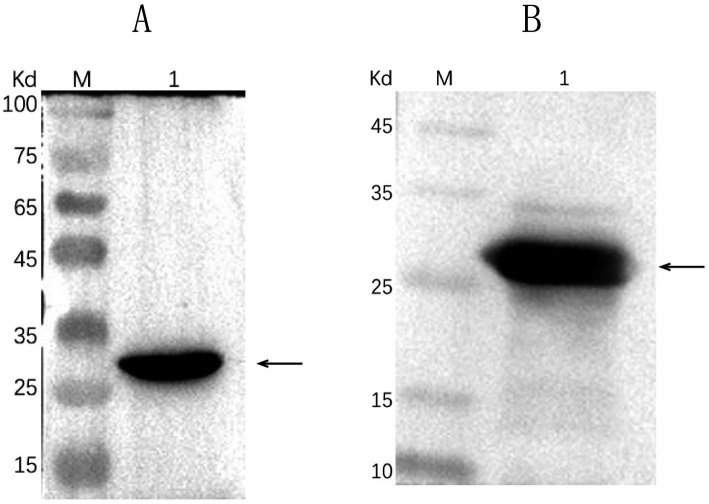
Western blot analysis of the △Cap fusion protein. M, protein molecular weight marker. **(A)** Lane 1, purified fusion protein blot incubated with mouse anti-His tag monoclonal antibody. **(B)** Lane 1, purified fusion protein blot incubated with prepared rabbit polyclonal antibody. The arrows pointed band are the expected protein.

Successful insertion of the optimized ΔCap gene sequence into the prokaryotic expression vector pET-30a (+) was confirmed by double digestion (Figure 1). The recombinant Cap protein was expressed as an inclusion body formation with a partially soluble protein with a molecular weight of approximately 27 kDa, as demonstrated by SDS-PAGE (Figure 2A). After optimizing the induction conditions, the highest yield of the △Cap protein was achieved with 0.5 mM IPTG at 37°C for 5 h (Figures 2B,C). The inclusion body protein was purified using a Ni-Agarose His tag protein purification kit, and this method yielded a purer protein sample compared to gel purification (Figures 3A,B). The immunoreactivity of the △Cap protein was subsequently assessed via Western blotting (Figure 4A). Further Western blot assays using rabbit polyclonal antibodies prepared with the recombinant protein also confirmed the immunogenicity and reactogenicity of the △Cap protein (Figure 4B).

### Development and evaluation of the △cap-iELISA

The purified △Cap protein was utilized as a diagnostic antigen for the development of an indirect ELISA method. The optimal coating antigen concentration determined by checkerboard titration was 4 μg/mL, with the optimal serum dilution being 1:100 (Table 1). Other optimized reaction conditions included coating the antigen at 37°C for 2 h and using a blocking solution of 2% skim milk powder at 37°C for 1 h. The HRP labeled goat anti-duck IgG (H + L) was diluted to 1:1000. The reaction times for serum, HRP labeled goat anti-duck IgG (H + L), and TMB were set at 60, 30, and 10 min, respectively according to the results of optimal tests (Supplementary Tables S1–S3). The mean OD value at 450 nm in 13 negative goose serum samples was 0.052, with a standard deviation of 0.032. Thus, the cut-off value (mean + 3 SD) for determining the state of serum samples in ELISA was 0.147.

**Table 1:** The determination of optimal coating conditions and secondary antibody dilution results of △Cap-iELISA.

Concentration of △Cap protein (μg/mL)	Constituencies	Serum dilution			
		1:50	1:100	1:200	1:400
0.5	P/N	11.313	18.041	31.350	44.467
	P	0.741	0.442	0.314	0.334
	N	0.066	0.025	0.010	0.008
1	P/N	10.626	17.380	33.278	46.067
	P	0.781	0.435	0.300	0.346
	N	0.074	0.025	0.009	0.008
2	P/N	9.685	14.968	17.026	31.696
	P	0.814	0.472	0.324	0.365
	N	0.084	0.032	0.019	0.012
3	P/N	11.600	16.718	30.500	49.611
	P	1.044	0.594	0.427	0.447
	N	0.090	0.036	0.014	0.009
4	P/N	10.029	**13.546**	26.051	31.750
	P	1.565	**1.030**	0.769	0.762
	N	0.156	**0.076**	0.030	0.024

Bold fonts represent values obtained under optimal conditions.

**Figure 5:**
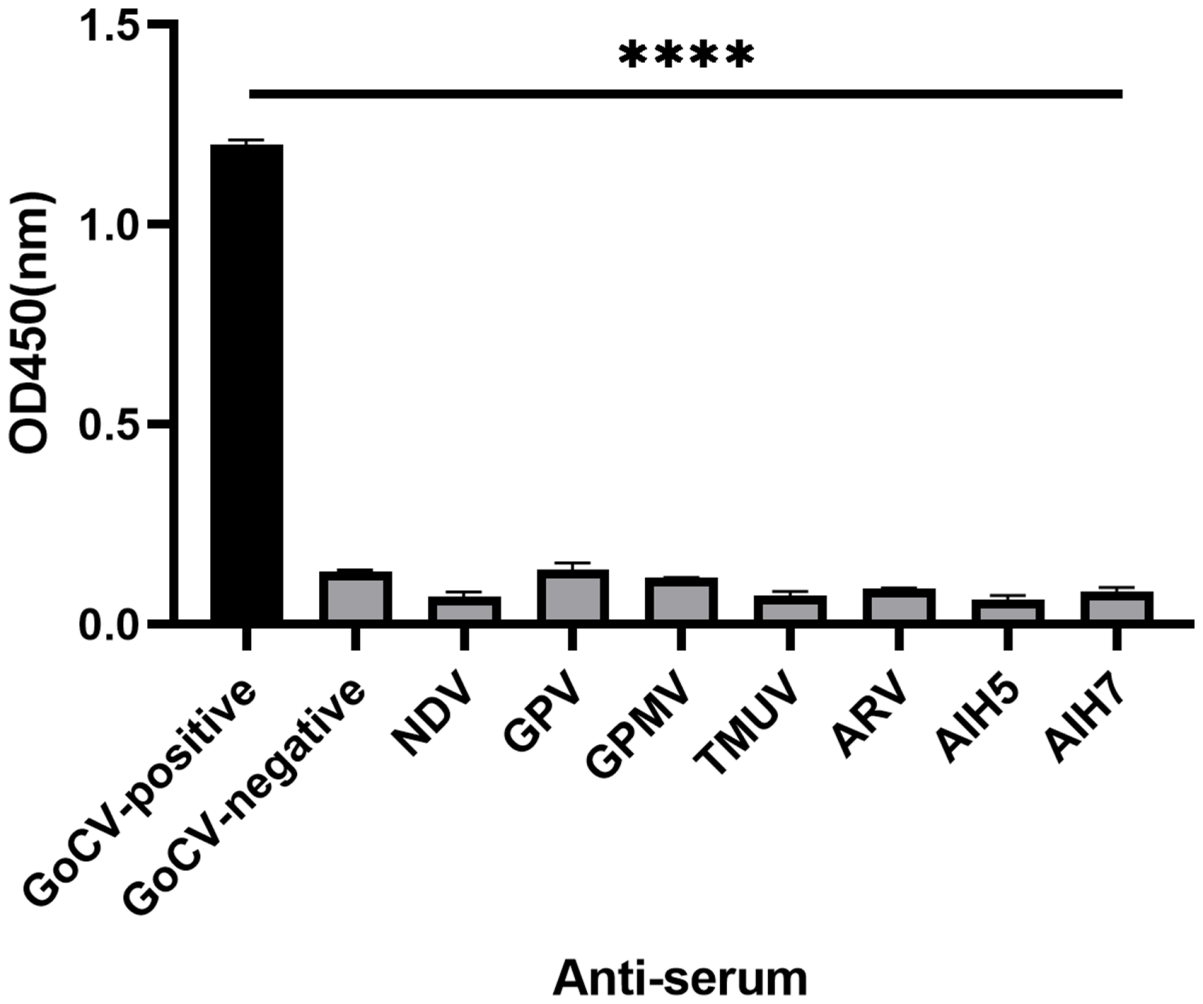
The specificity analysis of △Cap-iELISA against the common pathgens in geese. The reference anti-serum samples included GoCV positive anti-serum (GoCV-positive), GoCV negative anti-serum (GoCV-negative), Newcastle disease virus positive anti-serum (NDV), Goose parvovirus positive anti-serum (GPV), Goose paramyxovirus positive anti-serum (GPMV), Tembusu virus positive anti-serum (TMUV), Avian reovirus positive anti-serum (ARV) and H5 and H7 subtypes of Avian influenza virus positive anti-serum (AIH5, AIH7). **** for *p* < 0.0001.

The ELISA detection method was utilized for the detection of NDV, GPV, GPMV, ARV, AIV(H5), AIV(H7), and TMUV positive serum, Controls for GoCV positive and negative serum were also established. Except for the GoCV-positive serum, the OD450 nm values of the remaining sera below the cut-off value (Figure 5), indicating that the ELISA detection method established in this study did not cross-react with antibodies induced by common pathogens in geese. The established △Cap-iELISA was applied to six serum samples, with a coefficient of variation of the OD450 nm value in the batch ranging from 4.72 to 7.90%, and a coefficient of variation of the inter-batch replicate value ranging from 1.98 to 8.06% (Table 2). All coefficients of variation were below 10%, indicating that the ELISA detection method established in this study did not cross-react with antibodies induced by common pathogens in geese.

**Table 2:** The repeatability analysis results of △Cap-iELISA.

Sample number	Repeat within the batch						Batch-to-batch duplication					
	1	2	3	Average	Standard deviation	Coefficient	1	2	3	Average	Standard deviation	Coefficient
1	0.895	0.936	0.993	0.941	0.049	5.24%	0.881	0.923	0.971	0.925	0.045	4.89%
2	0.710	0.643	0.692	0.636	0.035	5.45%	0.597	0.589	0.612	0.599	0.012	1.98%
3	0.819	0.809	0.878	0.789	0.037	4.72%	0.789	0.799	0.905	0.831	0.064	7.75%
4	0.528	0.552	0.582	0.508	0.027	5.33%	0.508	0.540	0.553	0.534	0.023	4.35%
5	0.260	0.248	0.279	0.262	0.016	6.00%	0.249	0.259	0.269	0.259	0.010	3.74%
6	0.166	0.171	0.192	0.176	0.014	7.90%	0.166	0.159	0.185	0.170	0.014	8.06%

### Comparison of △cap-iELISA and real-time quantitative PCR (RT-qPCR)

One hundred and eighteen serum samples randomly selected from total samples were analyzed using △Cap-iELISA and confirmed by Real-time Quantitative PCR (RT-qPCR). Of these, 78 samples tested positive with △Cap-iELISA, while 48 tested positive by RT-qPCR. The Kappa value of 0.48835 for the two diagnostic methods indicated moderate consistency between the △Cap-iELISA assay and the RT-qPCR assay (Table 3).

**Table 3:** Comparison of the △Cap-iELISA with the Real-time Quantitative PCR.

		Real-time Quantitative PCR		
		Positive	Negative	Total
△Cap-iELISA	Positive (+)	47	31	78
	Negative (−)	1	39	40
	Total	48	70	118

### Field sample application

A total of 349 clinical serum samples were tested using the △Cap-iELISA method (Table 4). The results revealed that 248 out of 349 samples tested positive for GoCV antibodies, resulting in a high positive rate of 71.06%. Among these, sera from 163 breeding geese were examined, with 150 testing positive for the antibodies, yielding a positive rate of 92.02% (150/163). Additionally, 88 meat goose, day age between 40d and 100d, serum samples were analyzed, and 71 were positive for the antibodies, resulting in a high positive rate of up to 80.68% (71/88). Of the 98 gosling serum samples tested, only 27 were positive for the antibodies. Moreover, the positive rates of GoCV antibodies in breeding geese, meat geese, and gosling in Guangdong province were 96.24% (128/133), 86.20% (50/58), and 33.33% (16/48) respectively. These rates were significantly higher than those observed in Fujian and Shandong provinces at 73.33% (22/30), 70.00% (21/30), and 22.00% (11/50), respectively. The overall positive rate of GoCV antibodies in goose serum samples from Guangdong was higher than in samples from Fujian, and both were higher than in samples from Shandong (81.17, 76.67, and 22%, respectively).

**Table 4:** Clinical serum sample test results.

Age group	Region											
	Guangdong			Fujian			Shandong			Total		
	Amount of total samples	Amount of positive samples	Positivity rate %	Amount of total samples	Amount of positive samples	Positivity rate %	Amount of total samples	Amount of positive samples	Positivity rate %	Total number of samples	Number of positive samples	Positivity rate %
<40d	48	16	33.33	–	–	–	50	11	22.00	98	27	27.55
40–100d	58	50	86.20	30	21	70.00	–	–	–	88	71	80.68
>100d	133	128	96.24	30	22	73.33	–	–	–	163	150	92.02
Total	239	194	81.17	60	43	76.67	50	11	22.00	349	248	71.06

## Discussion

Studying the pathogenesis of GoCV has been challenging due to the lack of virus isolation and culture technology. Currently, research on GoCV primarily focuses on epidemiological investigation. The main methods for detecting GoCV are nucleic acid techniques, including routine PCR (14), nested broad-spectrum PCR (22), TaqMan real-time PCR, competitive PCR, dual PCR, Loop-mediated isothermal amplification (LAMP) (23), dot blot hybridization tests (DBH) (11), *in situ* hybridization (ISH) (24), and indirect fluorescent assays (IFA) (12). Serological assays, in contrast, are relatively rare. Therefore, it is necessary to establish an effective serological test to monitor GoCV and control the spread of the virus.

As the primary structural protein of GoCV, the cap protein contains five main B-cell antigenic epitopes (16) that may inducing an immune response and eliciting antibodies against the Cap protein in the body. Serological tests based on a recombinant capsid protein could serve as a valuable tool for epidemiological investigation. Studies have demonstrated that the entire Cap gene could not be expressed in *E. coli* due to the presence of NLS (25). Therefore, similar to beak feather disease virus (BFDV) (26), pigeon circovirus (PiCV) (27), and duck circovirus (DuCV) (28), We prepared truncated GoCV coat protein prokaryotic expression vectors, and subsequently, rabbit polyclonal antibodies were generated from the gel-purified proteins and their reactivity was assessed by Western blot analysis. The results showed that the purified target proteins maintained acceptably good immunogenicity and reactivity.

The ELISA detection method offers several advantages, including high sensitivity, strong specificity, good repeatability, and fast and convenient operation (29). Importantly, only a small volume of goose serum is required for detection. Our established ELISA method can complete antibody detection within 2 h when using the coated antigen, allowing for rapid results. Additionally, GoCV is often found alongside multiple pathogens such as TMUV and GPV (6, 18, 29); therefore, an assessment of cross-reactivity is necessary. The OD450 nm values of all groups except for GoCV antibodies were below the cut-off value, indicating the relatively good specificity of this △Cap-iELISA for GoCV antiserum (Figure 5). In comparison to the qPCR assay, the kappa value was 0.48835, indicating moderate consistency (Table 3). Repeatability tests also demonstrated that this method has good repeatability and is suitable for large-scale clinical trials.

The positive rate of GoCV antibodies in breeding and meat geese in Guangdong Province was significantly higher than that in Fujian Province. The positive rate of GoCV antibodies in goslings was also higher in Guangdong Province compared to Shandong Province. Overall, the positive rate of GoCV antibodies in goose serum samples from Guangdong Province is higher than that in Fujian Province, which is further higher than that in Shandong Province. Admittedly, due to the variation in the difficulty of sampling among different regions, the number of serum samples collected from geese at each age stage was not the same. However, even when the number of serum samples collected from goslings in Guangdong and Shandong was equal, the positive rate of GoCV antibodies remained higher in gosling serum from Guangdong compared to Shandong. This suggests a higher probability of goose flocks being infected with GoCV in Guangdong compared to other provinces. A similar trend of infection was observed among different goose breeds and age groups across Guangdong, Fujian, and Shandong provinces. The positive rate of GoCV serum antibodies increased gradually with the age of the geese. This finding aligns with research conducted by Scott et al. indicating that susceptibility to infection varies among geese of various breeds and age groups. In addition, our test detected a seroprevalence rate exceeding 80% (194/239) for geese from Guangdong, while our previous studies reported nucleic acid positivity rates exceeding 50% for goose flocks within this region (6). This indicates that GoCV infection is widely prevalent in Guangdong Province, China and that it is more severe compared to other provinces, even becoming an endemic disease among the geese raised in Guangdong. The above results suggest that △Cap-ELISA can be applied for large-scale clinical serological diagnosis of GoCV. Furthermore, China is the world’s largest goose producer. Given the high prevalence of GoCV infection in some parts of China, combined with the economic losses from growth retardation and feather dysplasia caused by GoCV infection in geese as well as the difficulties in disease prevention and control caused by co-infection and immunosuppression, it is necessary to develop effective diagnostic tests.

In conclusion, the △Cap-iELISA method has been successfully employed for the detection of antibodies to GoCV in geese. The method demonstrated high sensitivity, specificity, and reproducibility. The serological data of GoCV in goose flocks from Guangdong, Fujian, and Shandong were obtained through the detection of clinical samples. This study lays the groundwork for seroepidemiological investigations in these areas and provides valuable reference data for establishing standardized control measures.

## Data Availability

The datasets presented in this study can be found in online repositories. The names of the repository/repositories and accession number(s) can be found in the article/Supplementary material.
