# Supplementary material for: Epidemiological investigation of goose circovirus based on a newly developed indirect ELISA method
Source: Front Vet Sci. 2025 Feb 13;12:1521705. doi: 10.3389/fvets.2025.1521705 (PMC11866481; doi:10.3389/fvets.2025.1521705)
Supplement: Supplementary file 1 [file Table_1.pdf]

Supplementary file——

Epidemiological Investigation of Goose Circovirus Based on a Newly Developed Indirect ELISA Method

Jialong Chen[1], Zhanxin Yao[1], Wenchang Xue[1], Chao Wang[1], Wanjun Zhu[2], He Wang[1, 5], Jipei Zhang[1], Yi Tang[3], Rongchang Liu[4], Jidang Chen[1, 6*]

[1]School of Animal Science and Teachnology, Foshan University, Foshan, Guangdong, China, 528225
[2]WANMUZHOU Biotechnology Limited Company, Foshan, Guangdong, China, 528225
[3]College of Animal Science and Veterinary Medicine, Shandong Agricultural University, Taian, Shandong, China, 271018
[4]Institute of Animal Husbandry and Veterinary, Fujian Academy of Agricultural Sciences, Fuzhou, Fujian, China, 350013
[5]SHENZHEN KINGKEY SMART AGRICULTURE TIMES CO., LTD, Shenzhen, Guangdong, China, 518000
[6]Agricultural Teaching and Research Bases, Foshan University, Foshan, Guangdong, China, 528225

The Codon optimization of the △Cap gene sequence was showed as below.The codon optimization was based on the preference of E. coli.The underlined bases are the digestion sites, and the bold italics are the histidine tags:

CATATG***CACCACCACCACCACCAC***TCAAAGTACACTATATTCAACGTAAAGCAGACCCAGAAC
ATTAGCTTTACCTTCTTCGGCACCGGCAGCCCGGACAAGAACAAATGGCAGGCGATGAGCCTG
GAGGCGATCCAAAGCAGCGGTACCAGCCCGAAGCCGGGCATTAACCTGCGTTTCGCGGTGTTT
GGCGACCGTCTGCCGGGTACCGGTAACCAGTACCACTATCCGTTCGATTACTATATGATCCGTC
TGGTGAAGGTTGAACTGCGTCCGGCGTTCAACCCGTTTACCCGTGTTCGTACCCAAGGTAGCAC
CTACATCGACAAAGAGGGCAACATTACCACCACCACCAGCGGTAGCGAATGGAACGTGGATCC
GTACGCGGCGATGAGCAGCCGTAAAACCTGGAGCCCGCACCGTTATCACAAGCGTGTTTTCAT
CCCGAAACCGACCATTCAGCAAGGTGGCACCGGTAGCAACATCTGGAGCACCTGGTATACCCC
GGGTGGCCGTCAGCTGTGGCTGAACAGCATTCAAGACAACGTGGTGTTCTACGGTATGGGCAT
GAGCCTGCGTCAGGCGGAGGATACCGCGGCGCCGCTGACCGTGGAAGCGACCATCACCTACTA
CATCCGTTTTGGTCAATGGACCGGCCTGGCGCCGTAATGA

Table 1 The results for the determining optimal encapsulation conditions and secondary antibody dilution of △Cap-iELISA.

Coating conditions	Constituencies	Secondary antibody dilution				
		1:1000	1:2000	1:5000	1:8000	1:10000
4°C overnight	P/N	8.798	8.503	9.088	4.696	23.071
	P	1.329	0.702	0.364	0.216	0.323
	N	0.151	0.083	0.040	0.046	0.014
37°C 1h	P/N	14.085	18.475	15.649	10.676	21.438
	P	1.155	0.545	0.290	0.182	0.172
	N	0.082	0.030	0.019	0.017	0.008
37°C 2h	P/N	**10.233**	7.944	6.366	4.656	4.394
	P	**1.054**	0.564	0.357	0.217	0.218
	N	**0.103**	0.071	0.056	0.047	0.050
37°C 4h	P/N	15.814	21.074	10.132	13.588	8.356
	P	1.360	0.717	0.385	0.231	0.188
	N	0.086	0.034	0.038	0.017	0.023

Note: Bold fonts represent values obtained under optimal conditions.

Table 2 The results for the determining optimal antigen containment conditions of △Cap-iELISA.

Containment conditions	Constituencies	Containment fluid	
		2 % skimmed milk powder	5 % skimmed milk powder
4 °C, overnight	P/N	7.278	5.893
	P	1.714	2.175
	N	0.236	0.369
37 ℃, 30 min	P/N	7.644	5.271
	P	1.804	1.421
	N	0.236	0.270
37 ℃, 1 h	P/N	**5.468**	5.359
	P	**0.993**	1.016
	N	**0.182**	0.190
37 ℃, 2 h	P/N	4.189	4.481
	P	1.274	1.109
	N	0.304	0.248

Note: Bold fonts represent values obtained under optimal conditions.

Table 3 The results for the determining the optimal reaction time for serum and secondary antibodies of △Cap-iELISA.

Secondary antibody reaction time	Constituencies	Serum reaction time			
		15 min	30 min	45 min	60 min
37 ℃, 10 min	P/N	22.781	18.125	16.837	14.672
	P	0.365	0.435	0.362	0.426
	N	0.016	0.024	0.022	0.029
37 ℃, 15 min	P/N	18.145	16.451	13.072	12.319
	P	0.563	0.749	0.634	0.715
	N	0.031	0.046	0.049	0.058
37 ℃, 20 min	P/N	18.673	16.224	14.463	13.941
	P	0.514	0.617	0.579	0.711
	N	0.028	0.038	0.040	0.051
37 ℃, 25 min	P/N	17.638	14.827	15.000	13.438
	P	0.609	0.727	0.758	0.860
	N	0.035	0.049	0.051	0.064
37 ℃, 30 min	P/N	16.778	16.865	14.134	**12.621**
	P	0.755	0.877	0.898	**1.067**
	N	0.045	0.052	0.064	**0.085**

Note: Bold fonts represent values obtained under optimal conditions.
